# Apoptin-Armed Oncolytic Adenovirus Triggers Apoptosis and Inhibits Proliferation, Migration, Invasion, and Stemness of Hepatocellular Carcinoma Hep3B Cells

**DOI:** 10.3390/v17121636

**Published:** 2025-12-17

**Authors:** Zhaoxing Sun, Wenjie Li

**Affiliations:** Institute of Virology, Wenzhou University, Wenzhou 325000, China; microszx@163.com

**Keywords:** apoptin, liver cancer cells, recombinant adenovirus, cell apoptosis

## Abstract

Hepatocellular carcinoma (HCC) is a major cause of cancer-related mortality, highlighting the urgent need for novel therapeutic strategies. Apoptin, encoded by the VP3 gene of the chicken anemia virus, selectively induces apoptosis in cancer cells while sparing normal cells. We previously engineered a recombinant oncolytic adenovirus (Ad-VP3) capable of high-level Apoptin expression in tumor cells. In this study, we evaluated the antitumor activity of Ad-VP3 in the human HCC cell line Hep3B. CCK-8, crystal violet, Hoechst 33342 staining, flow cytometry, and tumor sphere formation assays revealed that Ad-VP3 inhibited cell viability, proliferation, and stemness. Annexin V staining, JC-1/TMRM probes, and Western blot analysis demonstrated induction of apoptosis and reduction of mitochondrial membrane potential. Wound-healing, Transwell, and BioCoat invasion assays, along with Western blotting, confirmed suppression of migration and invasion. Ad-VP3 significantly inhibited the viability, proliferation, migration, and invasion of Hep3B cells in a time- and dose-dependent manner. It induced mitochondrial membrane potential loss and apoptosis, downregulated stemness-related proteins (ALDH1A1, KLF4, and Sox2), and suppressed epithelial–mesenchymal transition markers (Snail, Twist1, Slug, Vimentin, and MMP-9), indicating strong antitumor activity. The recombinant oncolytic adenovirus Ad-VP3 exerts potent antitumor effects on hepatocellular carcinoma cells by inducing mitochondrial dysfunctionmediated apoptosis and impairing stemness and metastatic potential, suggesting its promise as a novel therapeutic strategy for HCC.

## 1. Introduction

Hepatocellular carcinoma (HCC) is the most common type of primary liver cancer, accounting for approximately 80% of cases, and remains the sixth most prevalent malignancy and the third leading cause of cancer-related deaths worldwide, according to the World Health Organization’s 2022 global cancer statistics [1]. The relative 5-year survival rate for HCC is only ~18%, reflecting its high lethality. Current therapeutic options for HCC are stage-dependent. In very-early- or early-stage disease, surgical resection, liver transplantation, and local ablative therapies offer the greatest chance of cure [2,3]. However, due to the asymptomatic nature of early HCC, most patients present at intermediate or advanced stages, when curative interventions are no longer feasible. At these later stages, transarterial chemoembolization (TACE) or radioembolization (TARE) can provide disease control in intermediate disease, while advanced disease is mainly treated with systemic molecular-targeted therapies such as sorafenib [4]. Nevertheless, despite continuous advances in conventional therapies, the high postoperative recurrence rate (often exceeding 50% within 2 years) and the emergence of drug resistance severely limit long-term survival [5], underscoring an urgent need for novel therapeutic approaches with improved efficacy and selectivity.

Oncolytic virotherapy (OV) has emerged as a promising anticancer strategy, using wild-type or genetically engineered viruses to selectively replicate within and lyse tumor cells without harming normal cells [6,7]. Among OV platforms, oncolytic adenoviruses (oAds) are the most extensively studied in clinical trials, representing approximately 42% of all OV-related trials. Their clinical safety profile has been well documented, with Oncorine—approved in China in 2005—being the first oAd to reach clinical use [8]. oAds offer several advantages, including strong induction of antitumor immunity, antiangiogenic effects, high-level transgene expression, and synergistic potential when combined with chemotherapy, radiotherapy, or immunotherapy [9].

Apoptin, encoded by the VP3 gene of the chicken anemia virus (CAV), is a small tumor-selective apoptosis-inducing protein with a unique mechanism of action: it triggers apoptosis through a p53- and Bcl-2-independent but caspase-dependent pathway, allowing it to effectively kill tumor cells with p53 mutations or loss of function—frequent events in HCC—while sparing normal cells [10,11]. This selectivity is largely attributed to its subcellular localization: in normal cells, Apoptin is retained in the cytoplasm and is non-toxic, whereas in tumor cells, it translocates to the nucleus and activates apoptotic cascades [12]. Compared to other pro-apoptotic agents, Apoptin’s independence from common apoptotic escape mechanisms makes it an attractive candidate for inclusion in oncolytic virotherapy platforms [10,13]. Apoptin can promote the phosphorylation of Nur77, triggering its translocation from the nucleus to the mitochondria. The C-terminal ligand-binding domain (LBD) of Nur77 interacts with the BH3 domain of Bcl-2, inducing a conformational change that disrupts the Bcl-2/Bax heterodimer [10,14]. This facilitates Bax oligomerization and mitochondrial outer membrane permeabilization (MOMP), leading to the release of cytochrome c (Cyt-c) and apoptosis-inducing factor (AIF) [15,16]. Within promyelocytic leukemia nuclear bodies (Pml-NBs), Apoptin associates with the sequestered anaphase-promoting complex/cyclosome (APC/C) to induce apoptosis; however, it can also efficiently induce apoptosis in cells with reduced or absent Pml expression [17,18]. Apoptin-mediated apoptosis also involves Bcl-2–dependent activation of the apoptotic protease activating factor-1 (Apaf-1) apoptosome [14,19]. Upon release, Cyt-c binds to Apaf-1, leading to apoptosome assembly, activation of caspase-9 and caspase-3, and ultimately the execution of apoptosis. In addition to its pro-apoptotic function, Apoptin can inhibit tumor cell proliferation by perturbing the cell cycle, causing arrest at the G2/M phase. Through interactions with multiple proteins, Apoptin directly or indirectly modulates the function of cell cycle regulators, thereby enforcing G2/M arrest [10]. Specifically, Apoptin binds to protein kinase C β1 (PKCβ1) and activates it, which in turn phosphorylates Apoptin [10,20]. This phosphorylation not only promotes its nuclear accumulation but may also regulate the phosphorylation state of other cell cycle-related proteins, contributing to cell cycle control.

**Figure 1:**
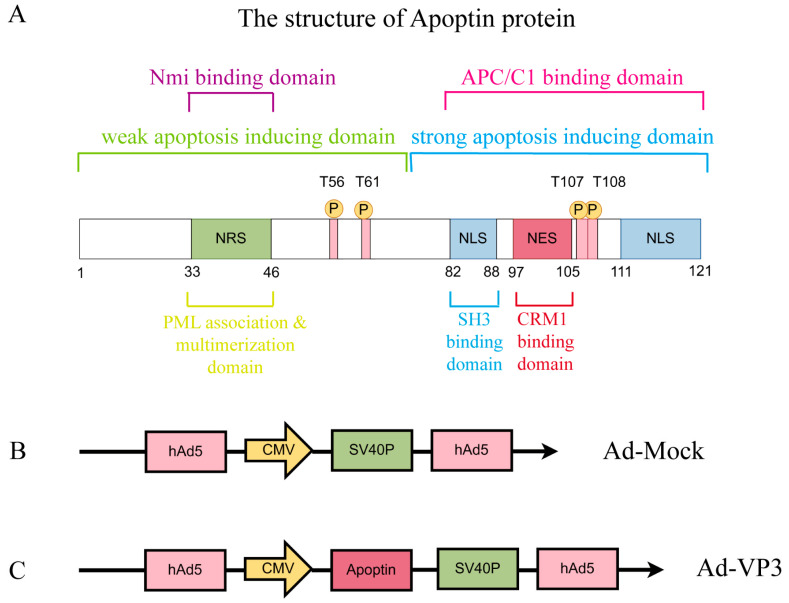
Diagram of Apoptin Protein Structure and the Two Recombinant Adenovirus Vectors Constructed in Our Laboratory Using Shuttle Vectors. These domains work synergistically to perform its function. (**A**) The structure of Apoptin: The C-terminal domain (amino acids 74–121) contains a bifunctional nuclear localization signal (NLS), a nuclear export signal (NES), and a tumor-specific phosphorylation site at T108. The NLS consists of two basic regions (NLS1: residues 82–88, NLS2: residues 111–121), ensuring efficient nuclear localization. The NES, located at residues 97–105, is recognized by CRM1 in normal cells, promoting cytoplasmic export. However, phosphorylation of T108 inhibits the NES function, enhancing nuclear accumulation. The N-terminal domain (amino acids 1–73) contains a polymerization center (residues 29–69) and a nuclear retention signal (NRS, residues 33–46). The polymerization center mediates monomer aggregation through a β-hairpin structure, while the NRS cooperates with the NLS to maintain nuclear retention. Additionally, the N-terminal region (residues 1–28) may prevent nuclear localization in normal cells by interacting with cytoplasmic proteins. (**B**) Ad-MOCK, (**C**) Ad-Apoptin (Ad-VP3).

Adenoviruses are among the most efficient gene delivery systems, possessing large genome capacity, high packaging titers, and ease of production, which together facilitate robust transgene expression with reduced risk of insertional mutagenesis compared to integrating vectors [9]. In our previous work, we constructed the recombinant oncolytic adenovirus Ad-VP3 based on a human adenovirus type 5 backbone, placing the Apoptin gene under the control of the strong CMV promoter to ensure robust expression in tumor cells [21]. The replication-defective adenovirus used in this study (E1a-deleted) Ad-VP3, as well as the empty control vector Ad-Mock, are schematically shown in Figure 1A. Ad-VP3 has demonstrated potent inhibitory effects in several tumor types, including lung, breast, and colorectal cancers [22,23,24]. Accumulating evidence indicates that cancer stem cells (CSCs) play a pivotal role in tumor initiation and progression due to their enhanced tumorigenic capacity and resistance to conventional anticancer therapies [25]. Therefore, novel cancer therapies should be evaluated not only for their ability to induce tumor regression but also for their efficacy in targeting CSCs. In our previous study, we showed that Ad-VP3, which induced tumor regression in an immunodeficient mouse xenograft model of the human breast cancer cell line MCF-7, was also capable of effectively targeting and eliminating CSCs within these tumors [22].

In this study, we sought to investigate the antitumor potential of Ad-VP3 in human HCC cells, using the Hep3B cell line as a model. We systematically evaluated its effects on cell viability, proliferation, and stemness through CCK-8, crystal violet, Hoechst 33342 staining, CFSE-based proliferation assays, and tumor sphere formation assays. Apoptotic induction and mitochondrial membrane potential (MMP) changes were examined by Annexin V staining, JC-1 and TMRM probes, and Western blotting. Furthermore, the impact of Ad-VP3 on migration and invasion was assessed using wound-healing assays, Transwell migration and BioCoat invasion models, alongside analysis of EMT- and stemness-related protein expression. This comprehensive approach not only evaluates Ad-VP3’s antitumor efficacy in HCC but also explores its potential mechanisms of action, providing a rationale for further preclinical development.

## 2. Materials and Methods

### 2.1. Cell Lines and Culture Conditions

Hep3B cells were obtained from the Institute of Virology, Wenzhou University. Cells were maintained in Minimum Essential Medium (MEM; Gibco, Shanghai, China) supplemented with 15% fetal bovine serum (FBS; Hyclone, Logan, UT, USA), 100 U/mL penicillin, and 100 mg/mL streptomycin (Hyclone, Logan, UT, USA). Cultures were incubated at 37 °C in a humidified atmosphere containing 5% CO_2_. The recombinant adenoviruses Ad-VP3 and Ad-Mock were constructed at the Molecular Virology and Immunology Laboratory, Military Veterinary Research Institute, Academy of Military Medical Sciences, Changchun, and preserved at the Institute of Virology, Wenzhou University. The titer of the recombinant adenovirus is expressed in plaque-forming units (PFU), calculated as:

PFU = (number of plaques × dilution factor)/volume of virus inoculum. In infection experiments, viral dose is expressed as the multiplicity of infection (MOI), which refers to the ratio of the number of infectious viral particles to the number of target cells at the time of infection, with units of PFU per cell (PFU/cell).

### 2.2. CCK-8 Assay

Hep3B cells were seeded into 96-well plates at a density of 5 × 10^3^ cells per well in 200 μL medium. An additional cell-free group served as a blank control. After 12–24 h incubation at 37 °C with 5% CO_2_, cells were treated with Ad-Mock or Ad-VP3 at multiplicities of infection (MOIs) of 100, or 200 (5 × l0^5^ PFU, 1 × l0^6^ PFU), and cultured for 24–96 h. At 24, 48, 72, and 96 h post-infection, 10 μL of CCK-8 solution (Cell Counting Kit-8 purchased from Tongren, Dojindo Laboratories, Kumamoto, Japan) was added to each well under light-protected conditions and incubated for 4 h. Absorbance was measured at 450 nm using a microplate reader.

The cell viability calculation formula is:

Cell viability (%) = [(Experimental well OD value − Blank well OD value)/(Control well OD value − Blank well OD value)] × 100%

### 2.3. Crystal Violet Staining Assay

Hep3B cells were seeded into 6-well plates at a density of 5 × 10^5^ cells per well in 2 mL medium and incubated for 12–24 h at 37 °C with 5% CO_2_. Cells were then treated with Ad-Mock or Ad-VP3 at MOIs of 100, or 200 (5 × l0^7^ PFU, 1 × l0^8^ PFU), and cultured for 24–96 h. At each time point (24, 48, 72, and 96 h), the medium was removed, and cells were rinsed with PBS, fixed with 4% paraformaldehyde, and stained with crystal violet (4% paraformaldehyde and crystal violet staining solution purchased from Beyotime, Shanghai, China). Images were captured under a microscope for analysis.

### 2.4. Hoechst 33342 Staining Assay

Hep3B cells were seeded into 6-well plates at 3.5 × 10^5^ cells per well in 2 mL medium and incubated for 12–24 h. Three groups were established: Ad-VP3, Ad-Mock, and untreated control. Cells were treated with 100 MOI (3.5 × 10^7^ PFU) of Ad-VP3 or Ad-Mock, and at 24, 48, and 72 h post-infection, Hoechst 33342 dye (10 μL) (purchased from Beyotime, Shanghai, China)was added to each well. After 10 min incubation at 37 °C in the dark, cells were washed twice with PBS and examined using a fluorescence microscope.

### 2.5. CFSE Staining Assay

Hep3B single-cell suspensions were labeled with CFSE (CFDA SE dye purchased from Beyotime, Shanghai, China) and seeded into 6-well plates at a density of 3.5 × 10^5^ cells per well in 2 mL medium. Staining efficiency was confirmed by flow cytometry prior to infection. Cells were then divided into Ad-VP3, Ad-Mock, and control groups, and infected with 100 MOI (3.5 × 10^7^ PFU) of Ad-VP3 or Ad-Mock. At 24, 48, and 72 h post-infection, cells were harvested, resuspended in 500 μL PBS, and analyzed by flow cytometry to assess cell proliferation.

### 2.6. Cell Cycle Analysis

Hep3B cells were seeded into 6-well plates at 3.5 × 10^5^ cells per well in 2 mL medium and incubated for 12–24 h. Cells were assigned to Ad-VP3, Ad-Mock, and control groups, and treated with 100 MOI (3.5 × 10^7^ PFU) of Ad-VP3 or Ad-Mock. At 24, 48, and 72 h post-infection, cells were harvested, resuspended in 500 μL of Vybrant^®^ DyeCycle™ Orange stain (purchased from Invitrogen, Waltham, MA, USA) (prepared by diluting 1 μL Vybrant^®^ DyeCycle™ Orange stain dye in 1 mL PBS), and incubated at 37 °C for 30 min. Cell cycle distribution was analyzed by flow cytometry.

### 2.7. Tumor Sphere Formation Assay

Hep3B cells were seeded in 12-well plates at a density of 2 × 10^5^ cells (1ml/well) and incubated at 37 °C with 5% CO_2_ for 12–24 h. The cells were then treated with 100 MOI (2 × 10^7^ PFU) of recombinant adenovirus Ad-VP3. Control groups included the Ad-Mock virus group and a blank control group. At 24, 48, and 72 h post-treatment, single-cell suspensions of Hep3B cells were collected, and live cell counts were performed. Subsequently, 2000 cells per well were plated into untreated 6-well plates. The cells were cultured in serum-free DMEM/F12 medium supplemented with 20 ng/mL bFGF, 20 ng/mL EGF, 2% B27, 1% N-2, and 1% penicillin-streptomycin (EGF, B27, and N-2 purchased from Invitrogen, Waltham, MA, USA; bFGF purchased from PeproTech, Rocky Hill, NJ, USA). Cells were observed every two days, and images were taken approximately one week after plating.

### 2.8. Annexin V Analysis

Hep3B cells were collected and seeded in 6-well plates at a density of 3.5 × 10^5^ cells per well in 2 mL medium. The plates were incubated at 37 °C with 5% CO_2_ for 12–24 h. The experimental groups, including the Ad-VP3, Ad-Mock, and control groups, were established. In each group, Hep3B cells were treated with 100 MOI (3.5 × 10^7^ PFU) of Ad-VP3 or Ad-Mock. At 24, 48, and 72 h post-treatment, one 6-well plate from each group was collected, and cells were dissociated into a single-cell suspension. The cells were resuspended in 500 μL of 1× Binding Buffer (from the Annexin V-FITC/PI Kit) and stained using the Annexin V-FITC/PI staining kit (purchased from BD Biosciences, Franklin Lakes, NJ, USA). Each sample was incubated with 5 μL of Annexin V-FITC and 5 μL of PI, and the samples were gently mixed. The cells were then incubated in the dark at room temperature for 15 min. After staining, cell apoptosis levels were assessed using both flow cytometry and fluorescence microscopy.

### 2.9. TMRM Staining Assay

Hep3B cells were collected and seeded in 6-well plates at a density of 3.5 × 10^5^ cells per well in 2 mL medium and incubated at 37 °C with 5% CO_2_ for 24 h. Subsequently, Ad-VP3 and Ad-Mock were added at a multiplicity of infection (MOI) of 100 (3.5 × 10^7^ PFU). At 24, 48, and 72 h post-infection, one 6-well plate from each group was collected, and cells were dissociated into a single-cell suspension. The cells were then incubated with 500 μL of TMRM dye, diluted at a 1:500 ratio (1 μL of TMRM and 500 μL of TMRM detection buffer), and incubated in the dark at 37 °C for 45 min. After incubation, the cell slides were inverted and observed under a fluorescence microscope. Following this treatment, the cell samples were transferred to flow cytometry tubes and analyzed using a flow cytometer.

### 2.10. JC-1 Staining Assay

JC-1 dye (purchased from Life Technologies, Carlsbad, CA, USA) was used to assess both qualitative and quantitative changes in mitochondrial membrane potential (MMP). Hep3B cells were collected and seeded in 6-well plates at a density of 3.5 × 10^5^ cells per well in 2 mL medium and incubated at 37 °C with 5% CO_2_ for 24 h. Subsequently, Ad-VP3 and Ad-Mock were added at a multiplicity of infection (MOI) of 100 (3.5 × 10^7^ PFU). At 24, 48, and 72 h post-infection, one 6-well plate from each group was collected, and cells were dissociated into a single-cell suspension. The cells were then incubated with 1 mL of JC-1 dye diluted at a 1:5000 ratio (0.2 μL JC-1 dye and 1 mL culture medium) and incubated in the dark at 37 °C for 15 min. After incubation, the cell slides were inverted and observed under a fluorescence microscope. Following this treatment, the cell samples were transferred to flow cytometry tubes and analyzed using a flow cytometer.

### 2.11. Western Blot

Hep3B cells were collected and seeded in 6-well plates at a density of 3.5 × 10^5^ cells per well in 2 mL medium. The plates were then incubated at 37 °C with 5% CO_2_ for 12–24 h. The experimental groups included the Ad-VP3 group, Ad-Mock group, and control group. In each group, Hep3B cells were treated with 100 MOI (3.5 × 10^7^ PFU) of Ad-VP3 or Ad-Mock. At 24, 48, and 72 h post-treatment, one 6-well plate was collected from each group, and the cells were harvested. Total protein was extracted using the Minute™ Total Protein Extraction Kit (purchased from Invent Biotechnologies, Eden Prairie, MN, USA). To each well, 200 μL of pre-chilled SC-001 cell lysis buffer (pre-added with protease and phosphatase inhibitors) was added. The cells were thoroughly lysed by grinding with a 1 mL pipette tip on ice. After lysis for 3–5 min on ice, the lysate was transferred to a pre-chilled column from the Invent kit. The sample was then centrifuged at 14,000 rpm for 1 min at 4 °C, and the column was discarded. The liquid in the collection tube was collected as the total protein solution. The protein concentration was determined using the BCA Protein Assay Kit (purchased from Beyotime Biotechnology, Shanghai, China). Protein quantification, concentration determination, and sample preparation were then performed. Western blotting was used to analyze the expression levels of Apoptin and apoptosis-related proteins, including Cleaved-caspase3, Caspase3, PARP, Survivin, and Bcl-2. In addition, migration and invasion-related proteins such as Slug, Vimentin, Snail, Twist1, and MMP-9, as well as stemness-related proteins ALDH1A1, KLF4, and SOX2, were also analyzed. All Western blot results were normalized to GAPDH and quantified relative to the control group. The anti-Apoptin antibody was purchased from Abcam (Cambridge, UK). The antibodies for Cleaved-caspase3, PARP, Caspase3, Survivin, GAPDH, Bcl-2, Snail, Slug, Twist1, Vimentin, MMP9, ALDH1A1, KLF4, and Sox2 were purchased from Cell Signaling Technology (Danvers, MA, USA).

### 2.12. Wound Healing Assay

Hep3B cells were seeded at a density of 5 × 10^5^ cells per well in a 6-well plate with 2 mL per well and cultured in a 37 °C, 5% CO_2_ incubator for 12–24 h. When the cells reached 80–90% confluence, three parallel lines were scratched in the center and on both sides of the well bottom using the tip of a 200 μL pipette, creating a rectangular area devoid of cells. The area of the rectangular scratch was recorded by capturing images under a microscope (denoted as 0 h). After discarding the original culture medium, serum-free medium was added, followed by the addition of 20 MOI (1 × 10^7^ PFU) of recombinant adenovirus Ad-VP3 and Ad-Mock. Four hours later, MEM medium containing 2% serum was added. At 12 and 24 h post-infection, one 6-well plate from each group was selected. The same area photographed at 0 h was located under the microscope, and images were captured to calculate the change in scratch area.

The cell migration rate was calculated as:

Cell migration rate = (0 h scratch area − 24 h scratch area)/0 h scratch area × 100%

### 2.13. Transwell Migration and Invasion Assay

Hep3B cells were collected and seeded at a density of 5 × 10^5^ cells per well in a 6-well plate with 2 mL per well, and cultured in a 37 °C, 5% CO_2_ incubator for 12–24 h. Three groups were set up: Ad-VP3, Ad-Mock, and Control. Afterward, 100 MOI (5 × 10^7^ PFU) of Ad-VP3 and Ad-Mock were added to the respective wells. At 24, 48, and 72 h post-treatment, cells were collected from one 6-well plate at each time point. Next, at 24, 48, and 72 h post-infection, a new 24-well plate was prepared. To the lower chamber of each well, 500 μL of 10% DMEM medium was added, while the upper chamber was seeded with 200 μL of cell suspension at the same cell density. After 24 h of incubation, non-invasive cells in the upper chamber were removed with a sterile cotton swab. In the dark, 60 μL of CCK-8 reagent was added to the lower chamber, and the Transwell and Biocoat chambers (Transwell Permeable Supports, Matrigel Matrix purchased from Corning, Corning, NY, USA) were reinserted into the wells. The plate was incubated at 37 °C in the dark for 4 h. After incubation, the 24-well plate was removed, and the chambers were taken out. The OD value at 450 nm was measured using a microplate reader. The chambers were then stained with crystal violet, and the number of cells that had migrated through the membrane was quantified by capturing images under a microscope.

### 2.14. Statistical Processing

Each experiment was repeated three times or more. The obtained data were analyzed using GraphPad Prism 10 software, and all data are expressed as the mean ± standard deviation. Statistical comparisons between two groups were performed using a *t*-test, while comparisons among three or more groups were conducted using one-way analysis of variance (ANOVA). A *p*-value > 0.05 indicates no statistical significance, * *p* < 0.05 indicates statistical significance, ** *p* < 0.01, and *** *p* < 0.001 indicate highly significant differences.

## 3. Results

### 3.1. Ad-VP3 Exerts Cytotoxic and Growth-Inhibitory Effects on Hep3B Cells

**Figure 2:**
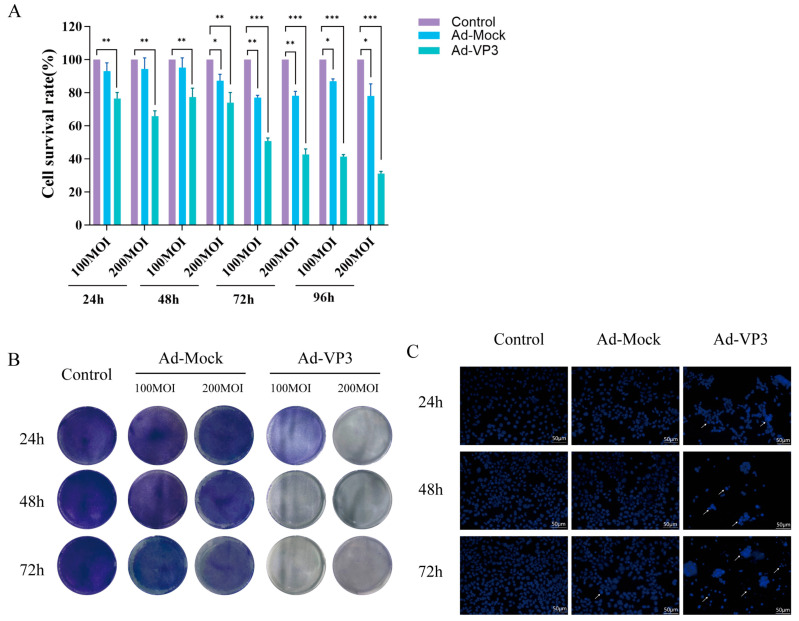
Cytotoxic and growth-inhibitory effects of Ad-VP3 on Hep3B cells. (**A**) CCK-8 assay results showing time- and dose-dependent cytotoxicity of Ad-VP3 at 24, 48, 72, and 96 h post-infection. (**B**) Crystal violet staining of Hep3B cells infected with Ad-VP3 or Ad-Mock at MOIs of 100, or 200, followed by staining at 24, 48, and 72 h. (**C**) Hoechst 33342 staining reveals apoptotic nuclear morphology following treatment with 100 MOI of Ad-VP3. White arrows indicate nuclear condensation and fragmentation revealed by Hoechst 33342 staining, characteristic of apoptotic cells.Data are mean ± SD (* *p* < 0.05, ** *p* < 0.01, *** *p* < 0.001).

To evaluate the in vitro antitumor activity of recombinant adenovirus Ad-VP3, Hep3B cells were infected with varying doses of Ad-Mock or Ad-VP3. CCK-8, a water-soluble tetrazolium salt, is reduced by dehydrogenases in cells to form a stable orange-yellow formazan product. Therefore, its absorbance value is strictly linearly correlated with the number of viable cells. CCK-8 assays revealed that Ad-VP3 significantly inhibited Hep3B cell viability compared with Ad-Mock (*p* < 0.05), in a dose- and time-dependent manner (Figure 2A). Crystal violet staining, a quantitative method for assessing cell proliferation, clearly and intuitively reflects the dynamic changes in the viable cell population. Crystal violet staining further confirmed that Ad-VP3 produced a stronger inhibitory effect than Ad-Mock (Figure 2B). Given that Apoptin, encoded by the VP3 gene of the chicken anemia virus, can induce apoptosis in tumor cells, the more pronounced cytotoxicity of Ad-VP3 is consistent with its pro-apoptotic potential. Hoechst 33342 staining was used for apoptosis-specific morphological analysis. This fluorescent dye can penetrate intact cell membranes and bind specifically to DNA, revealing characteristic nuclear morphological features. Hoechst 33342 staining showed that Ad-VP3 treatment markedly increased the proportion of cells exhibiting nuclear condensation and apoptotic bodies, indicating typical apoptotic morphology (Figure 2C).

### 3.2. Ad-VP3 Suppresses Proliferation and Self-Renewal of Hep3B Cells

**Figure 3:**
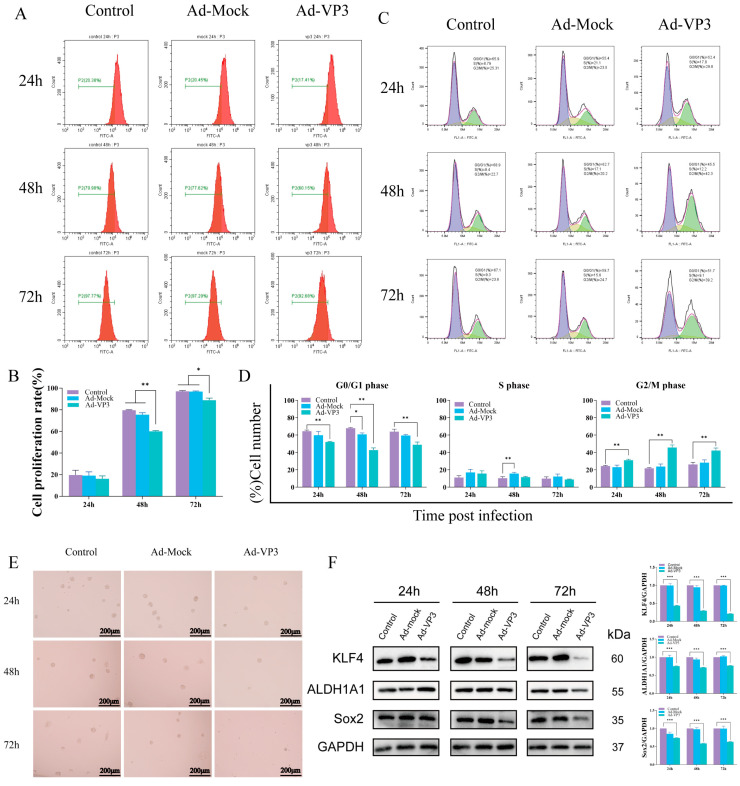
Inhibition of proliferation and stemness by Ad-VP3 in Hep3B cells. (**A**,**B**) CFSE-based flow cytometry showing reduced cell division in Ad-VP3-treated cells at 48 h and 72 h. (**C**,**D**) Cell cycle analysis showing G2/M arrest induced by Ad-VP3. In Figure 3C, the purple shaded area represents the G0/G1 phase, the yellow shaded area represents the S phase, and the green shaded area represents the G2/M phase. (**E**) Tumor sphere formation assay showing reduced sphere number and size after Ad-VP3 treatment (scale bar = 200 μm). (**F**) Western blot analysis showing downregulation of ALDH1A1, KLF4, and Sox2 in Ad-VP3-treated cells. All infections depicted in the figures were performed at a multiplicity of infection (MOI) of 100. Data are mean ± SD (* *p* < 0.05, ** *p* < 0.01, *** *p* < 0.001).

To assess the impact of recombinant adenovirus Ad-VP3 on the proliferation of Hep3B cells, we conducted a CFSE staining assay followed by flow cytometry analysis (Figure 3A,B). CFSE is a fluorescent dye that can penetrate cell membranes and enter cells, where it is hydrolyzed by intracellular esterases. The hydrolyzed CFSE molecules react with free amino groups in intracellular cytoskeletal proteins, ultimately forming fluorescent protein adducts. In flow cytometry detection, CFSE bound to the cytoskeleton emits green fluorescence upon excitation. As the parent cells divide, the fluorescent marker is equally distributed to the daughter cells, resulting in a logarithmic decay of fluorescence intensity. This allows for precise quantification of cell proliferation and division. The CFSE results showed that the fluorescence decay rate in the Ad-VP3-treated group at both 48 and 72 h was significantly lower than that in the control group (*p* < 0.05), indicating a marked inhibition of cell division. In contrast, there was no significant difference in the fluorescence decay curves between the Ad-Mock and control groups, suggesting that Ad-VP3 effectively inhibits Hep3B cell proliferation. Cell cycle analysis revealed that treatment with recombinant adenovirus Ad-VP3 induced a G2/M phase arrest in Hep3B cells, thereby inhibiting the proliferation of liver cancer cells (Figure 3C,D). Tumorsphere formation assays demonstrated that, under serum-free conditions for one week, Hep3B cells treated with Ad-VP3 formed fewer and smaller tumorspheres compared to the control or Ad-Mock groups (Figure 3E). Western blot analysis indicated that Ad-VP3 may inhibit the stemness of Hep3B cells by downregulating the expression levels of stemness-related proteins such as ALDH1A1, KLF4, and Sox2 (Figure 3F).

### 3.3. Ad-VP3 Induces Apoptosis in Hep3B Cells

**Figure 4:**
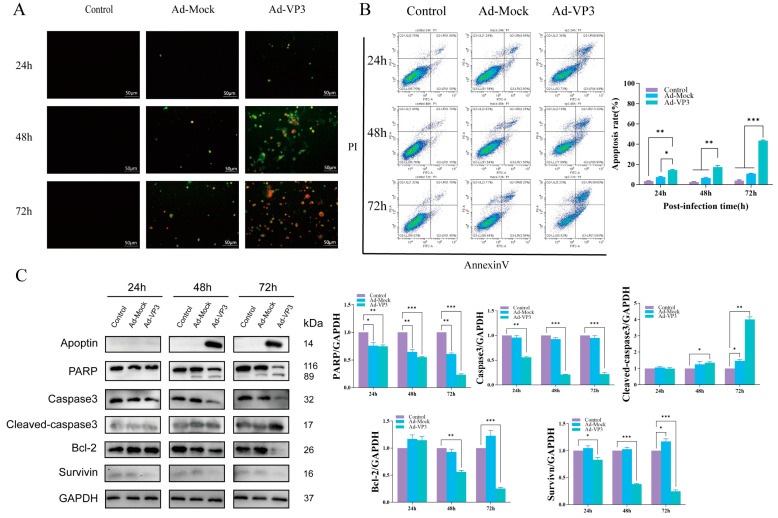
Apoptosis induction by Ad-VP3. (**A**) Annexin V-FITC/PI staining showing increased apoptotic morphology over time. At 24, 48, and 72 h post-infection with Ad-VP3 at 100 MOI, phosphatidylserine externalization and nuclear fragmentation increased markedly over time in the Ad-VP3 group. During apoptosis, phosphatidylserine exposure allows Annexin V–FITC binding (green fluorescence), while PI uptake in late apoptotic or necrotic cells produces red fluorescence. (**B**) Flow cytometric quantification of apoptotic cells. The apoptosis level in Hep3B cells infected with 100 MOI Ad-VP3 was significantly higher than that in the Ad-Mock and control groups. (**C**) Western blot analysis of Apoptin, PARP, Caspase-3, Cleaved caspase-3, Survivin, and Bcl-2 levels in Hep3B cells treated with 100 MOI recombinant adenovirus. Ad-VP3 effectively expressed apoptin. Scale bar = 50 μm. Data are mean ± SD (* *p* < 0.05, ** *p* < 0.01, *** *p* < 0.001).

To determine whether the recombinant adenovirus induces hepatocellular carcinoma cell death via apoptosis, we first evaluated apoptosis levels using Annexin V-FITC/PI staining (Figure 4A,B). During apoptosis, early and late apoptotic cells expose phosphatidylserine on the outer leaflet of the plasma membrane, which binds Annexin V in the Annexin V-FITC/PI kit and emits green fluorescence, while late apoptotic or necrotic cells take up PI and emit red fluorescence. Annexin V-FITC/PI staining showed that apoptotic cells exhibiting green and red fluorescence could already be observed in the recombinant adenovirus group at 24 h post-infection. At 48–72 h, the intensity and proportion of green and red fluorescent cells further increased, indicating a marked elevation in the apoptotic cell population (Figure 4A). Flow cytometry analysis revealed that the proportion of apoptotic cells increased progressively over time, with apoptosis rates in the Ad-VP3 group reaching approximately 15%, 19%, and 44% at 24, 48, and 72 h, respectively (Figure 4B). Western blot analysis confirmed Apoptin expression in the Ad-VP3 group. Notably, Apoptin protein was not detected at 24 h, although flow cytometry still showed a relatively high apoptosis rate in the Ad-VP3 group. This may be due to Apoptin expression interfering with cellular metabolism at the transcriptional or translational level, and/or a direct cytotoxic effect of adenoviral infection itself, both of which could trigger early apoptotic signaling. In the Ad-VP3-treated group, the expression level of cleaved caspase-3 was markedly upregulated, accompanied by cleavage of its substrate PARP. At the same time, Ad-VP3 significantly downregulated the expression of pro-caspase-3 and the anti-apoptotic proteins Bcl-2 and Survivin (Figure 4C). Together, these findings indicate that Ad-VP3 exerts a pronounced, time-dependent pro-apoptotic effect on Hep3B cells.

### 3.4. Ad-VP3 Induces Mitochondrial Membrane Potential Loss in Hep3B Cells

**Figure 5:**
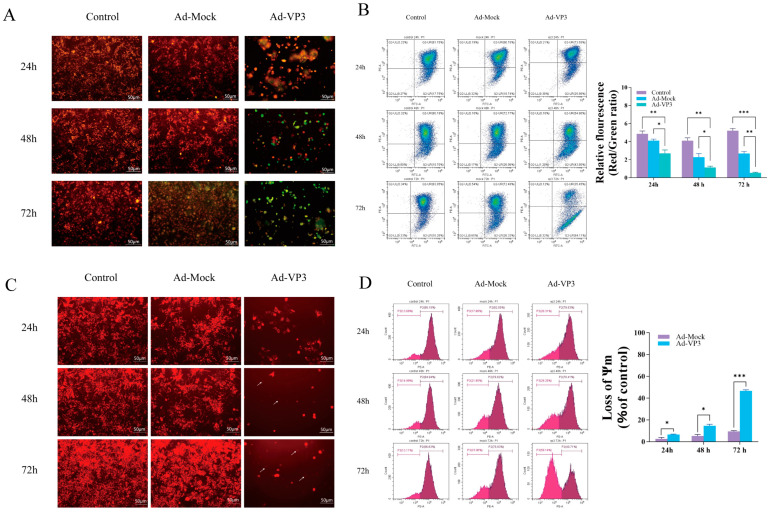
Effects of Ad-VP3 on mitochondrial membrane potential in Hep3B cells. (**A**,**B**) JC-1 staining showing time-dependent reduction in red/green fluorescence ratio. JC-1 detects mitochondrial membrane potential changes: polarized mitochondria show red fluorescence (JC-1 aggregates), whereas depolarized mitochondria during apoptosis display green fluorescence (JC-1 monomers). (**C**,**D**) TMRM staining confirming MMP loss in Ad-VP3-treated cells.White arrows indicate quenched TMRM fluorescence caused by decreased mitochondrial membrane potential.All infections depicted in the figures were performed at a multiplicity of infection (MOI) of 100. Scale bar = 50 μm. Data are mean ± SD (* *p* < 0.05, ** *p* < 0.01, *** *p* < 0.001).

To explore whether Ad-VP3 inhibits Hep3B cell growth via the intrinsic apoptotic pathway, we examined changes in the mitochondrial membrane potential (MMP) of Hep3B cells in vitro using JC-1 and TMRM staining. JC-1 is a fluorescent probe that alters its fluorescence characteristics in response to changes in mitochondrial membrane potential, allowing for the detection of MMP changes. When the mitochondrial membrane potential is intact, JC-1 accumulates in the mitochondrial matrix in a polymerized form, emitting red fluorescence. However, during apoptosis, when the mitochondrial membrane potential is depolarized, JC-1 cannot accumulate in the mitochondria and remains in its monomeric form, emitting green fluorescence. This property of JC-1 allows for the detection of mitochondrial membrane potential changes in cells. The JC-1 results demonstrated a significant decrease in MMP in the Ad-VP3-treated group after infection (Figure 5A,B). Over time, JC-1 fluorescence transitioned from red aggregates to green monomers, showing a time-dependent change (Figure 5A). Flow cytometry analysis revealed a significant decrease in the red/green fluorescence ratio in the Ad-VP3 group over time (*p* < 0.05). At 72 h, the red/green fluorescence ratio was at its lowest in the Ad-VP3 group, significantly lower than that in the Ad-Mock and control groups (*p* < 0.01) (Figure 5B). Next, we further analyzed the qualitative and quantitative changes in MMP in Hep3B cells using TMRM staining (Figure 5C,D). TMRM is a fluorescent dye that selectively accumulates on the mitochondrial inner membrane, with fluorescence intensity reflecting the state of the mitochondria. After entering the cells, TMRM is cleaved by intracellular esterases and then captured by mitochondria, binding to the mitochondrial inner membrane. When the mitochondrial membrane potential is normal, TMRM accumulates on the mitochondrial membrane, emitting orange-red fluorescence. However, when the mitochondrial membrane potential decreases, TMRM is released into the cytoplasm, resulting in fluorescence quenching. Therefore, the intensity of TMRM fluorescence can be used to assess the mitochondrial state. Fluorescence results (Figure 5C) showed that in the control group, Hep3B cells exhibited bright orange-red fluorescence, indicating normal MMP. In contrast, in the recombinant adenovirus group, due to decreased MMP, the orange-red fluorescence intensity was reduced and became completely transparent (indicated by white arrows). Flow cytometry results (Figure 5D) showed that, compared to the Ad-Mock group, Ad-VP3 treatment induced mitochondrial membrane depolarization in Hep3B cells. Moreover, with prolonged exposure, the number of cells with depolarized mitochondria was significantly higher in the Ad-VP3 group than in the Ad-Mock group. These results suggest that Ad-VP3 significantly reduces the mitochondrial membrane potential in Hep3B cells, inducing apoptosis. In conclusion, the inhibitory effect of Ad-VP3 on Hep3B cells is associated with changes in mitochondrial membrane potential (MMP), indicating that Ad-VP3 may induce apoptosis in Hep3B cells via the intrinsic apoptotic pathway.

### 3.5. Ad-VP3 Inhibits Migration and Invasion of Hep3B Cells

**Figure 6:**
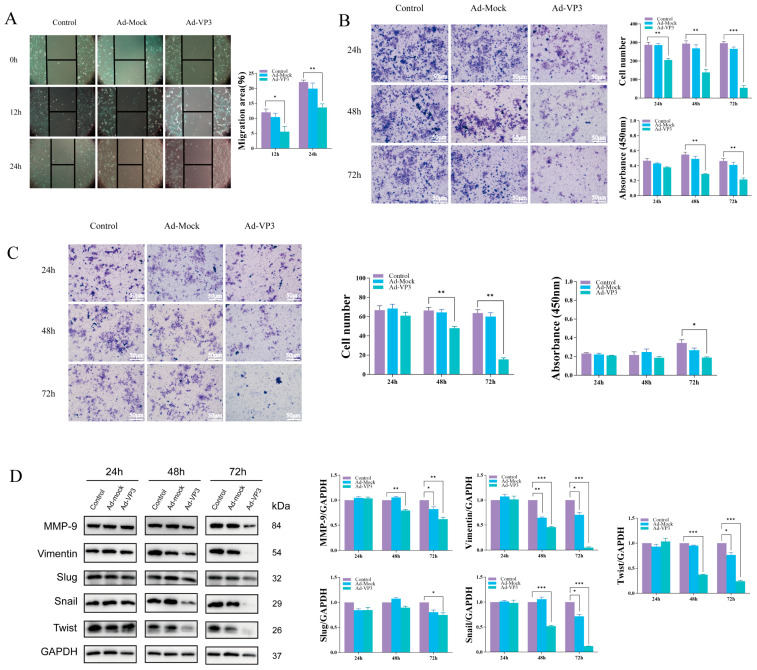
Inhibitory effects of Ad-VP3 on migration and invasion of Hep3B cells. (**A**) Wound-healing assay showing reduced migration rates at 12 h and 24 h. At 12 and 24 h after infection with 20 MOI of Ad-VP3, the wound healing area in the Ad-VP3 group was significantly reduced compared to the control group. (**B**,**C**) Transwell migration and BioCoat invasion assays showing decreased motility in Ad-VP3-treated cells. At 72 h post-infection with 100 MOI of Ad-VP3, the migration and invasion of Hep3B cells in the Ad-VP3 group were significantly reduced compared to the control group. (**D**) Western blot analysis showing downregulation of Snail, Twist1, Slug, Vimentin, and MMP-9. The virus dosage used in the WB experiment was 100 MOI. Scale bar = 200 μm. Data are mean ± SD (* *p* < 0.05, ** *p* < 0.01, *** *p* < 0.001).

We next examined whether Ad-VP3 affects the migratory and invasive capacities of Hep3B cells. Wound-healing assays demonstrated that cell migration was significantly reduced in the Ad-VP3 group compared with controls at both 12 h and 24 h (*p* < 0.01) (Figure 6A). Similarly, Transwell migration and BioCoat invasion assays showed that Ad-VP3 significantly suppressed migration and invasion compared with the control group (Figure 6B,C). The Transwell chamber assay uses a permeable membrane filter with 8 μm pores to create a dual-layer culture system, allowing cells with migratory potential to penetrate the filter membrane and migrate into the lower chamber under the influence of chemotactic factors. The Biocoat invasion assay is based on the Transwell assay, with the addition of Matrigel-coated filters to assess the malignant invasive characteristics of tumor cells. This experiment simulates the in vivo invasion process by coating the surface of the Transwell membrane with an extracellular matrix, such as laminin, to create a biophysical and biochemical barrier. Tumor cells with invasive potential must secrete matrix metalloproteinases (MMPs) to degrade extracellular matrix components, allowing them to penetrate the membrane and complete the invasion process. The number of cells that penetrate the membrane is proportional to the invasive activity of the cells. After pre-treatment with 100 MOI recombinant adenovirus Ad-VP3 and Ad-Mock, Hep3B cells were seeded in Transwell and Biocoat invasion chambers at 24, 48, and 72 h, and membrane penetration was observed by crystal violet staining. Under serum-induced conditions, cells in the control group at all time points showed partial membrane penetration. Although the number of cells that penetrated the membrane in the Ad-Mock group was slightly lower than that in the control group, a large number of cells still invaded. In contrast, the number of cells that penetrated the membrane in the Ad-VP3 group was significantly reduced, and this number decreased progressively with time. Western blot analysis revealed that Ad-VP3 downregulated the expression of epithelial–mesenchymal transition (EMT)- and invasion-associated proteins, including Snail, Twist1, Slug, Vimentin, and MMP-9 (Figure 6D), suggesting that Ad-VP3 impairs metastatic potential by modulating EMT-related pathways.

## 4. Discussion

Oncolytic virotherapy is being extensively developed in clinical settings, and adenoviruses were among the first gene therapy vectors evaluated in clinical trials, with their safety profiles widely assessed and documented [26,27]. Oncorine, approved in China in 2005, was the first oncolytic virus (OV) licensed for clinical use and serves as a landmark demonstration of the feasibility of oncolytic adenoviruses (oAds) in clinical application [8]. The major advantages of oAds include their ability to elicit potent antitumor immune responses, exert anti-angiogenic effects, achieve high transgene expression, and synergize with other therapeutic modalities. Notably, oAds are the most frequently used OV type in clinical trials, accounting for approximately 42% of all studies [26].

Building on this foundation, our group previously engineered the recombinant adenovirus Ad-VP3, based on a human adenovirus type 5 backbone, with the Apoptin gene under the control of a CMV promoter to enable robust expression of the tumor-selective apoptosis-inducing protein [21]. Using the human hepatocellular carcinoma cell line Hep3B as a model, we evaluated the cytotoxic, antiproliferative, and pro-apoptotic effects of Ad-VP3. Initial CCK-8 and crystal violet assays revealed that Ad-Mock exerted minimal inhibitory effects on cell viability, whereas Ad-VP3 displayed clear tumor-suppressive activity in a time- and dose-dependent manner. Hoechst 33342 staining further showed that Ad-VP3-infected cells exhibited nuclear condensation and fragmentation, indicative of apoptosis. CFSE-based flow cytometry demonstrated that Ad-VP3 markedly inhibited Hep3B cell proliferation, while Ad-Mock had no such effect. Further cell cycle analysis revealed that Ad-VP3 increased the proportion of cells in G2/M phase, inducing cell cycle arrest—findings consistent with similar observations in other cancer models [28]. Cancer stem cells (CSCs) play a central role in tumor initiation and progression. This subpopulation of cancer cells with stem cell-like properties is capable of self-renewal and differentiation into multiple cell lineages, and is critically involved in tumor onset, progression, therapeutic resistance, and metastasis [25,29]. With increasing evidence supporting the pivotal role of CSCs in cancer evolution and resistance to current therapies, the evaluation of novel treatment strategies must consider not only their ability to induce tumor regression but also their efficacy in targeting CSCs. With respect to stemness, Ad-VP3 exerted a marked inhibitory effect on both tumorsphere-forming capacity and the expression of stemness-associated proteins in Hep3B cells. We observed a pronounced reduction in the number of tumorspheres in the Ad-VP3 group, and a time-dependent decrease in the expression of the stemness markers ALDH1A1, KLF4, and Sox2 at 48 and 72 h post-infection. ALDH1A1 (aldehyde dehydrogenase 1 family member A1) is one of the most commonly used markers of CSCs; its high activity is closely associated with intracellular aldehyde metabolism, resistance to oxidative stress, and drug tolerance, and is often correlated with poor prognosis and increased risk of recurrence in various cancers [30]. KLF4 (Krüppel-like factor 4) is a transcription factor involved in maintaining stem cell pluripotency and self-renewal [31]. It is frequently overexpressed in CSCs and regulates cell cycle and differentiation through its downstream targets. Sox2 (SRY-box transcription factor 2) is another core stemness-related transcription factor that plays a key role in both embryonic stem cells and CSCs by sustaining an undifferentiated state and promoting proliferative potential [32]. These findings suggest that Ad-VP3 may inhibit the self-renewal and differentiation capacity of CSCs by modulating the expression of stemness-related proteins, thereby attenuating tumor malignancy and reducing the risk of recurrence. This is consistent with other studies targeting the regulation of CSC stemness and further supports the role of Ad-VP3 in suppressing CSC-like properties.

Regarding cancer stemness, Ad-VP3 significantly impaired sphere-forming capacity and reduced the expression of stemness-associated proteins ALDH1A1, KLF4, and Sox2 in a time-dependent manner at 48 h and 72 h post-infection. This suggests that Ad-VP3 may suppress tumor stem cell self-renewal and differentiation by downregulating key stemness regulators, thereby potentially reducing tumor aggressiveness and recurrence risk. These findings align with prior research on targeting cancer stem cell properties as a therapeutic strategy.

A hallmark of cancer is the combination of uncontrolled proliferation and evasion of apoptosis [33]. Apoptosis is essential for removing abnormal cells and maintaining tissue homeostasis, but tumor cells often escape this fate through diverse mechanisms, including downregulation of tumor suppressors (e.g., p53, PTEN) and overexpression of anti-apoptotic factors (e.g., Bcl-2, Bcl-XL, IAPs) [34]. The loss of apoptotic control confers survival advantages, allowing accumulation of genetic mutations that drive tumor progression, aggressiveness, and poor differentiation. For example, p53 plays a central role in preventing oncogenic transformation, and its tumor-suppressive function is partly mediated through apoptosis induction [35]. Loss of p53 function is a common selection during tumorigenesis. Similarly, overexpression of anti-apoptotic proteins such as Bcl-2 and Bcl-XL, often due to chromosomal translocations, contributes to cancer development and treatment resistance [36]. Many chemotherapeutic agents fail in tumors harboring p53 loss or overexpressed anti-apoptotic proteins. Apoptin is a tumor-selective apoptosis inducer that acts independently of p53 and Bcl-2, enabling it to kill tumor cells with p53 mutations or loss of function.

Previous studies have reported that Ad-VP3 exerts a significant inhibitory effect on HepG2 cells, with the apoptosis rate reaching 43.02% at 72 h [37]. It is known that HepG2 cells harbor wild-type p53, whereas Hep3B cells are p53-deficient [38]. Therefore, in the present study, we investigated whether the recombinant adenovirus Ad-VP3 could also induce apoptosis in p53-deficient Hep3B cells. In our study, AnnexinV-FITC/PI staining and flow cytometry confirmed that Ad-VP3 effectively induced apoptosis in Hep3B cells, with apoptotic cells evident early after infection and increasing markedly over time, reaching 44.25% at 72 h. This result stands in sharp contrast to the Ad-Mock group and strongly demonstrates that Ad-VP3 possesses p53-independent apoptosis-inducing activity. Loss of mitochondrial membrane potential (MMP) is a pivotal event in apoptosis. Using JC-1 and TMRM flow cytometry, we observed that Ad-VP3-induced MMP reduction correlated positively with treatment duration. This phenomenon suggests that Ad-VP3–induced apoptosis in Hep3B cells is most likely mediated through a mitochondria-dependent apoptotic pathway. MMP loss can lead to mitochondrial permeability transition pore opening, cytochrome c release, and activation of the downstream caspase cascade [34]. While Ad-Mock caused only transient and minimal MMP changes—likely representing a mild stress response—Ad-VP3 induced sustained mitochondrial disruption.

Western blot analysis provided molecular evidence for the apoptotic mechanism: Ad-VP3 upregulated the executioner caspase cleaved-caspase-3 and its substrate cleaved-PARP, while downregulating procaspase-3, the survival factor Survivin, and the anti-apoptotic protein Bcl-2. Cleaved caspase-3 is a defining step in apoptosis execution, and increased cleaved-PARP further corroborates apoptosis progression. The downregulation of Survivin and Bcl-2 suggests suppression of survival signaling and dismantling of anti-apoptotic defenses. Together, these findings indicate that Ad-VP3 induces apoptosis in Hep3B cells primarily via the intrinsic pathway through dual modulation of pro- and anti-apoptotic proteins.

Tumor metastasis is a complex, multistep process that severely impacts patient prognosis [39]. In this study, wound-healing, Transwell migration, and BioCoat invasion assays showed that Ad-VP3 significantly reduced Hep3B cell migration and invasion. Correspondingly, Western blotting revealed that Ad-VP3 downregulated EMT- and invasion-associated proteins, including Snail, Twist1, Slug, Vimentin, and MMP-9, in a time-dependent manner. Snail and Twist1 are key EMT transcription factors whose downregulation may restore epithelial marker expression (e.g., E-cadherin) and strengthen cell–cell adhesion, thereby reducing migratory and invasive potential. MMP-9, a matrix metalloproteinase that degrades extracellular matrix components, was also reduced, potentially limiting tumor cell dissemination [40,41,42]. In summary, Ad-VP3 inhibits migration, invasion, sphere formation, and stemness in Hep3B cells by downregulating proteins such as Snail, Twist1, MMP-9, ALDH1A1, KLF4, and Sox2.

This study has limitations. While we identified changes in key protein expression, the upstream regulatory pathways through which Ad-VP3 exerts these effects remain unclear. Future work should elucidate these signaling cascades and investigate potential intermediary regulators. Moreover, the current study was conducted exclusively in vitro; in vivo validation in animal models is essential to assess therapeutic efficacy and safety, thereby providing a more robust foundation for clinical translation. Despite these limitations, our findings provide strong theoretical support for further development of Ad-VP3 as a potential therapeutic agent for hepatocellular carcinoma and highlight its promise as a novel cancer treatment strategy.

## Data Availability

The data used to support the findings of this study are available from the corresponding author upon request.
